# Comparison of Multiple-Locus Variable-Number Tandem Repeat Analysis Profiles of Enteropathogenic *Yersinia* spp. Obtained from Humans, Domestic Pigs, Wild Boars, Rodents, Pork and Dog Food

**DOI:** 10.3390/ani13193055

**Published:** 2023-09-29

**Authors:** Axel Sannö, Thomas Rosendal, Anna Aspán, Annette Backhans, Magdalena Jacobson

**Affiliations:** 1 Department of Clinical Sciences, Swedish University of Agricultural Sciences, 750 07 Uppsala, Sweden; magdalena.jacobson@slu.se; 2 Department of Disease Control and Epidemiology, National Veterinary Institute, 751 89 Uppsala, Sweden; 3 Department of Animal Health and Antibiotics, National Veterinary Institute, 751 89 Uppsala, Sweden

**Keywords:** *Y. enterocolitica*, *Y. pseudotuberculosis*, multiple-locus variable-number tandem repeat analysis (MLVA)

## Abstract

**Simple Summary:**

Yersiniosis in humans is a gastrointestinal disease sometimes complicated by more severe symptoms with long-lasting consequences, such as reactive arthritis and erythematous nodosum. The identification of the source of this infection can be difficult due to several reasons. The food items responsible for the spread of the infection can be difficult to identify or have been consumed. Also, the cultivation of the bacteria *Yersinia (Y.) enterocolitica* and *Yersinia pseudotuberculosis* is laborious and difficult. Even after successful cultivation, comparison between different strains of the bacteria is not straightforward, and available techniques depend on access to the bacteria in a pure culture. In the present study, possible reservoirs of these bacteria, such as wild boars and pork, were compared using multiple-locus variable-number tandem repeat analysis (MLVA), showing that wild boars can be a possible source of yersiniosis. This technique offers the possibility of identifying possible reservoirs of contaminated foodstuff without the need for cultivation of the bacteria. This study also shows the presence of *Y. entercolitica* in minced meat from wild boars.

**Abstract:**

The enteropathogenic *Yersinia* genus is commonly detected in wildlife including wild boars. Difficulties in its cultivation may hamper subsequent epidemiological studies and outbreak investigations. Multiple-locus variable-number tandem repeat analysis (MLVA) of *Yersinia (Y.) enterocolitica* and *Y. pseudotuberculosis* has proven useful in source attribution and epidemiological studies but has hitherto relied on the analysis of isolates. In the present study, MLVA profiles generated from 254 isolates of *Y. enterocolitica* indicated similarities between human, pig and rodent isolates. Further, MLVA analyses of 13 *Y. pseudotuberculosis* pure-cultured isolates were compared to MLVA analyses performed directly on the 14 PCR-positive enrichment broths from which the isolates originated, which showed matching MLVA profiles. This indicates that MLVA analysis performed directly on enrichment broths could be a useful method for molecular epidemiological investigations. In addition, 10 out of 32 samples of wild boar minced meat obtained from private hunters and from approved wild-game-handling establishments were PCR-positive for the presence of *Y. enterocolitica* and may indicate a risk for public health.

## 1. Introduction

The enteropathogenic *Yersinia* genus is present in both domestic pigs and wild boar [1,2,3], and pork is suggested as the most probable source of infection in humans [4,5]. Common symptoms of acute yersiniosis include abdominal pain, vomiting and diarrhoea, and chronic sequelae such as reactive arthritis and erythema nodosum may occur. Yersiniosis is the third most prevalent enteric disease in Europe at an annual notification rate of 2.2 cases per 100,000 people, with Finland and Denmark reporting the highest rates of 10.64 and 9.54 cases per 100,000 people, respectively [6]. Calculations in Sweden indicated that for every reported case, an additional 15 cases remained undetected [7]. Further, young children seem more prone to contracting the infection since the notification rate for children less than six years of age is more than three times higher than the total notification rate [8].

All strains of *Yersinia (Y.) pseudotuberculosis* are considered pathogenic, while only certain bioserotypes of *Y. enterocolitica* have the potential to carry the virulence genes necessary to cause disease. Further, the pathogenicity of isolates carrying single virulence genes is debated [9]. The most commonly isolated bioserotype in patients suffering from yersiniosis is *Yersinia enterocolitica* 4/O:3 [10], whereas other bioserotypes, as well as cases of *Y. pseudotuberculosis*, are reported less frequently [11,12].

Source attribution and outbreak investigations on enteropathogenic *Yersinia* spp. have been limited by the diagnostic methods available [13]. However, in recent years, multiple-locus variable-number tandem repeat analysis (MLVA) protocols have been developed, which may bring further insight into its epidemiology [14,15]. Further, we recently developed a protocol utilizing enrichment broth to obtain MLVA profiles without the need for isolation of the bacteria [16]. This technique can be used in highly contaminated samples such as faeces and food [17] and allows the MLVA results to be obtained faster and with a higher sensitivity than those obtained using methods requiring isolation of the bacteria. MLVA profiles also offer the possibility of establishing a common database to compare data between different laboratories. Sporadic human cases may thus be linked to larger outbreaks and the potential sources of human disease can be ranked if enough data are available from humans and putative sources of *Yersinia* spp. [18,19].

The aim of this study was to compile MLVA profiles of presumptively pathogenic *Y. enterocolitica* from wild boar minced meat and various other sources, including humans, domestic pigs, rodents, wild boars and food stuffs, and to assess the population diversity via cluster analysis. Furthermore, MLVA profiles of *Y. pseudotuberculosis* obtained from Swedish wild boar were compared by sampling location.

## 2. Materials and Methods

### 2.1. Collection of Samples

Frozen packages of minced wild boar meat were bought in supermarkets and specialized farm shops selling wild-game meat. These products all originated from different approved game-handling establishments (AGHEs). Further, samples were obtained from private hunters as well as one game-handling establishment that submitted samples of wild boar minced meat upon request (an additional seven wild-game-handling establishments were contacted but did not respond). Whenever possible, the packages of retail minced meat were gathered from different slaughter batches. Private hunters were requested to sample minced meat during the processing of carcasses or submit already-frozen minced meat from their private freezer. The wild-game-handling establishment was instructed to sample 50 g of fresh wild boar minced meat once a week during a four-week period and send the samples on ice to the laboratory.

Further, a total of 207 isolates of enteropathogenic *Y. enterocolitica* originating from domestic pigs and wild boar from previous studies performed at SLU and the National Veterinary Institute were included for comparison (SVA) [1,16,20]. Thirteen isolates were also obtained from the Swedish Food Protection Agency (by courtesy of Susanne Thisted Lambertz), and thirty-four isolates from patients suffering from yersiniosis were obtained from the Public Health Agency of Sweden (by courtesy of Cecilia Jernberg). Furthermore, thirteen isolates of *Y. pseudotuberculosis* [16,21] and twelve enrichment broths (Brain Heart Infusion, BHI) that were PCR-positive for the presence of *Y. pseudotuberculosis* were included [16].

### 2.2. Laboratory Analysis

Five grams of wild boar minced meat was added to 45 mL of buffered peptone water (BPW), incubated on a slant for 20 ± 2 h at +28 °C to facilitate isolation of motile bacteria and analysed according to Sannö et al. [16]. Briefly, following incubation, 10 µL from the top layer of the broth was streaked on selective agar plates and incubated for 20 ± 2 h. DNA was extracted from colony material using an Instagene Matrix^®^ (BioRad, Hercules, CA, USA) according to the manufacturer’s instructions and analysed via PCR for the presence of *Y. enterocolitica* and *Y. pseudotuberculosis*. Primers and PCR conditions are presented in Sannö et al. [16].

All isolates of *Y. enterocolitica* and all PCR-positive enrichment broths obtained in the analysis of minced wild boar meat were analysed via MLVA [16]. Briefly, genomic DNA was purified (GeneJETGenomic DNA Purification Kit Fermentas, St. Leon-Rot, Germany) according to the manufacturer’s instructions, MLVA primers [22] were labelled with ABI PRISM^®^ fluorescent dyes, and the VNTR loci V2A (PET), V4 (NED), V6 (6-FAM), V5 (NED), V7 (VIC), and V9 (PET) were used in the analyses. Example of the electropherogram generated is presented in Figure S1. The reference strain CCUG 45,643 (4/O:3) was used as a positive control.

All isolates of *Y. pseudotuberculosis* were analysed using the MLVA method presented by Halkilahti, Haukka [23] with the forward primers labelled with a fluorescent 6-FAM dye and the reactions carried out to amplify the loci YPbF1, YPbF3, YPbF5, YPbF7, YPbF8, YPbF9 and YPbF10. All amplifications were carried out in a BioRad DNA Engine Dyad Peltier Thermal Cycler (Hercules, CA, USA). The results from the MLVA performed on enrichment broths demonstrated positivity according to PCR for *Y. pseudotuberculosis*, as obtained in a previous study using the same samples [16].

### 2.3. Population Diversity

To assess the ability of the MLVA protocol to describe the population diversity, Simpson’s index of diversity was calculated for all possible allele combinations using the vegan package [24] in R version 3.4.4 (Vienna, Austria) [25] in order to determine the relative contribution of each allele to the discriminatory power of the MLVA methods.

### 2.4. Cluster Analysis

**Figure 1:**
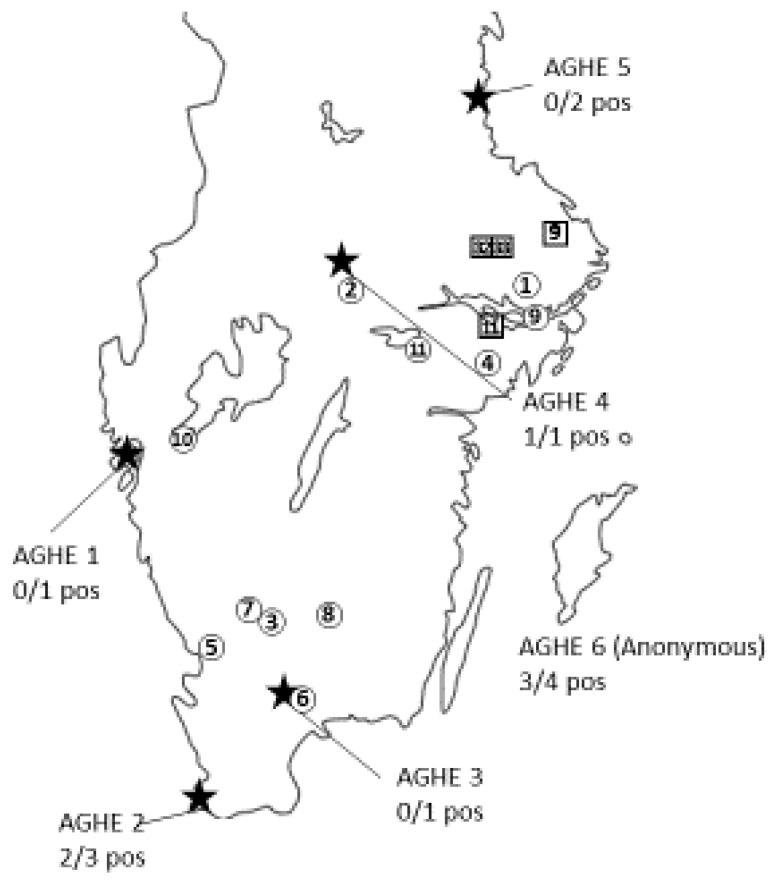
The locations of the wild-game-handling establishments are indicated by a star ★ (Est. 1–6). Minced meat submitted by these establishments was analysed via PCR for the presence of *Y. enterocolitica* and *Y. pseudotuberculosis.* Locations of private hunting areas positive for *Y. enterocolitica* according to PCR are indicated by circled numbers, while the sampling locations of samples positive for *Y. pseudotuberculosis* that also yielded MLVA profiles are indicated by a number within a square. The number ① given within each symbol refers to the consecutive numbering of each sample given upon arrival to the laboratory.

A dendrogram was constructed, utilizing the proportion of loci that differed between the isolates as the distance metric (Figure 1) as calculated with the “dist.gene” function in the “ape” package [26], followed by the building of a single-linkage cluster dendrogram also utilizing the ape package in R 4.0.3. (Vienna, Austria). Identical isolates were only included in the tree once, and the number of isolates of a given MLVA type was indicated by varying sizes of the end node, along with the bioserotype or sources of the isolates, which were indicated by different colours.

## 3. Results

**Figure 2:**
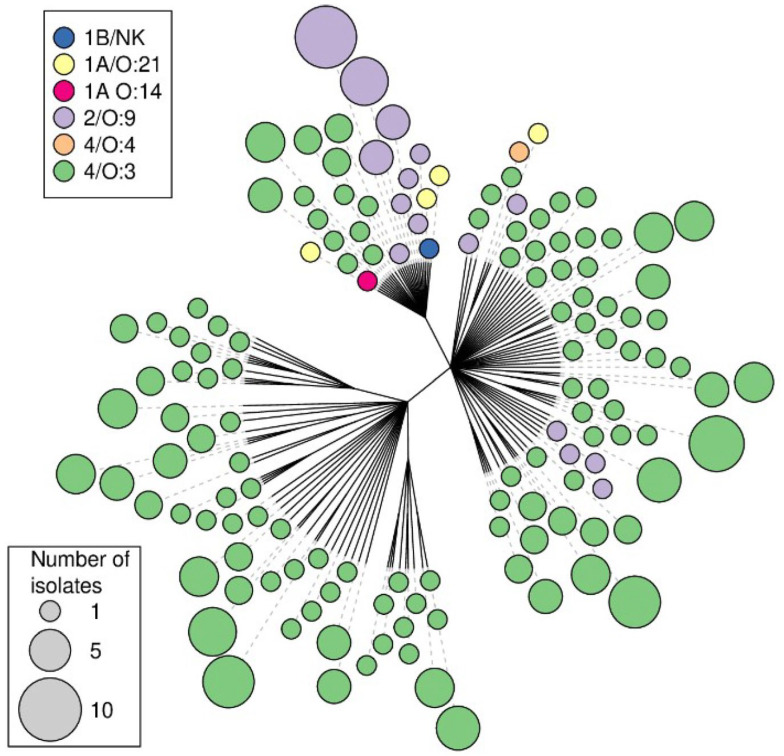
Unrooted Minimum Spanning Tree constructed on the basis of 145 MLVA profiles of *Y. enterocolitica* isolates. Each dot represents a single unique detected profile; the colour of the dot represents the bioserotype of the isolate, and the size of the dot represents the number of isolates with that unique MLVA profile.

In total, 12 wild boar minced-meat samples were obtained from six approved game-handling establishments (AGHEs), while 20 samples were obtained from private hunters (Table 1). The sampling locations are indicated in Figure 2. Ten of the samples (31.3%) were positive for *Y. enterocolitica* according to PCR performed on the BHI broth, including six samples from wild-game-handling establishments and four samples from private hunters. No isolates were obtained, and none of the samples were PCR-positive for *Y. pseudotuberculosis*. No single, complete MLVA profile was obtained for any of these samples, and the locus-specific results of the MLVA are presented in Table 2.

The final analysis of the MLVA profiles obtained from the *Y. enterocolitica* isolates (Table S1) included 203 isolates from domestic pigs, 34 isolates from humans with yersiniosis, 7 isolates from pork products, 3 isolates from wild boar, 1 isolate from dog food, 2 isolates from rodents and 4 isolates of unknown origin from an isolate collection at the National Food Agency. These 254 isolates displayed 145 unique MLVA profiles, of which 94 profiles originated from domestic pigs. Simpson’s index of diversity for all loci was calculated as 0.986 and for the individual loci V2A, V4, V5, V6, V7 and V9, the index of diversity was 0.916, 0.814, 0.910, 0.886, 0.913 and 0.359, respectively. One profile obtained from a pig was also identified in a rodent caught on the same farm (Figure 1). Single-repeat differences were seen in two human isolates of bioserotype 2/O:9 that matched with pig isolates in all loci apart from locus V2A. Furthermore, single-repeat differences were seen in loci V6 and V7 between two human isolates of the bioserotype 4/O:3 isolated one year apart. In the cluster dendrogram, which also displays the sources of the isolates, isolates of bioserotypes other than 4/O:3 grouped together (Figure 1).

**Figure 3:**
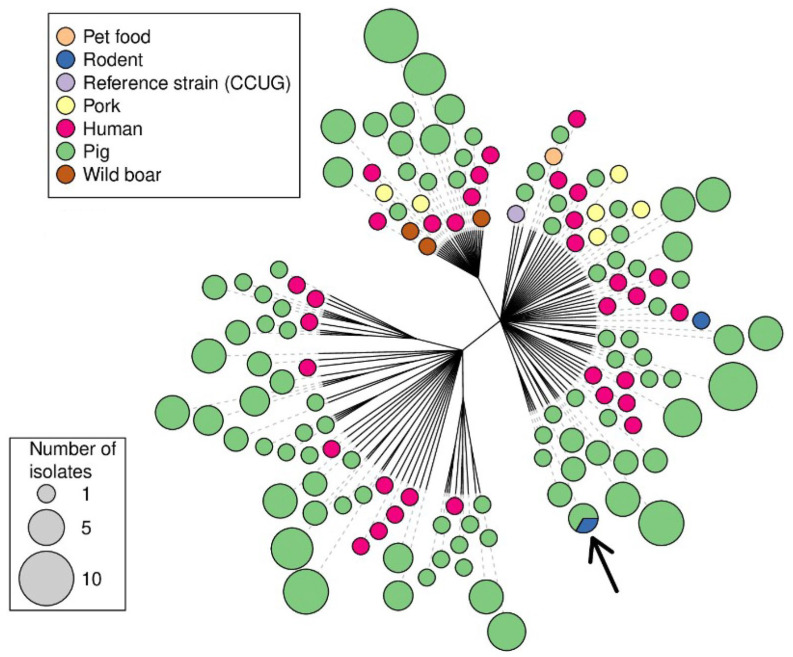
Unrooted Minimum Spanning Tree constructed from 145 MLVA profiles of *Y. enterocolitica* isolates. Each dot represents a single unique detected profile; the colour of the dot represents the origin of the isolate, and the size of the dot represents the number of isolates with that unique MLVA profile. The arrow indicates an identical profile obtained from pigs and a rodent sampled on the same farm.

The analysis of the 13 isolates of *Y. pseudotuberculosis* resulted in seven unique MLVA profiles (Table 3; Figure 2 and Figure 3). The MLVA of the 12 PCR-positive enrichment broths yielded 13 unique profiles, and 1 profile with information missing in one locus (Table 3; Figure 2 and Figure 3). Furthermore, in one sample, two PCR fragments were obtained from locus YPB3B (Table 3, animal no. 17), representing four and seven repeats, and in one sample, two PCR fragments were present for locus YPB7B (Table 3, animal no. 18), representing one and three repeats, respectively. Hence, these two samples displayed two possible MLVA profiles each. Identical MLVA profiles were obtained in the analysis of two isolates obtained following the cultivation of the BHI broth and in the analysis performed directly on the BHI enrichment broth (Table 3). Matching MLVA profiles obtained after analysis of the isolates were found in samples from different geographical sampling sites (Table 3; Figure 2 and Figure 3).

## 4. Discussion

Multiple-locus variable-number tandem repeat analysis (MLVA) has a high discriminatory power and is hence mainly useful when performed for outbreak investigations and source attribution studies [18]. This method has become the gold standard for such investigations in *Salmonella enterica* subsp. *enterica* and *Salmonella enterica* serovar Typhimurium. Common protocols and databases have been developed, proving useful tools for outbreak investigations of human salmonellosis [27]. Similarly, protocols have been developed for *Y. pseudotuberculosis* [23] and *Y. enterocolitica* [15,22].

In the present study, no perfect matches were found between the MLVA profiles obtained from patients and the isolates of other origin. However, as described in Virtanen et al. [28], allowing for single-repeat differences in the highly variable loci V2A, V5, V6 and V7 make MLVA a useful tool to detect similarities between isolates of different origins. The single-repeat differences seen in two human isolates matched pig isolates in all loci apart from the highly variable locus V2A and are thus in line with the understanding that most human cases of yersiniosis are of pig origin [4,5]. Likewise, the single-repeat differences seen in loci V6 and V7 between two human isolates of the bioserotype 4/O:3, isolated one year apart, may indicate a link between these cases. The assessment of the discriminatory power of each allele indicated that the inclusion of V9 did not increase the discriminatory power of the MLVA in this set of samples. However, since the presently investigated sample collection was limited, no definite conclusions should be drawn. The perfect match between isolates from pigs and rodents in the same farm (Figure 1) [20] supports the usefulness of the method and may indicate that rodents play a role in the epidemiology of *Y. enterocolitica* in pig farms, or that both pigs and rodents are infected from a common source on that farm.

MLVA has previously proven useful in epidemiological investigations [18] and in investigating potential outbreaks of yersiniosis in humans [15]. In the present study, 254 isolates of *Y. enterocolitica* from various sources were analysed and compiled into a database. It is evident that the non-4/O:3 isolates cluster together (Figure 2, left) and that no single MLVA type contains more than one bioserotype. This indicates that an MLVA profile, or a presumptive MLVA profile obtained after analysis of the enrichment broth [16], could be used to identify the bioserotype of an isolate. Further investigation is needed to determine the usefulness of MLVA as a classification method for enteropathogenic *Yersinia* spp. in epidemiological investigations. A larger investigation of the discriminatory power of this method in relation to the time of sampling, geographical location of the farms and other known connections between the farms is needed. However, the present study presents a foundation and indicates that MLVA may be useful in this respect.

*Y. enterocolitica* was demonstrated to be present in wild boar minced meat both from supermarkets and in meat obtained from private hunters. This indicates that contamination of the meat and the carcass during the slaughter process occurs, and furthermore, MLVA performed on enrichment broth from minced-meat samples indicated that more than one strain may be present in some samples. Hence, in contrast to the analysis of pure-culture isolates, the analysis of the primary enrichment broth may be useful to gain information on several strains possibly present in the sample. In the present study, multiple PCR fragments were present in various loci in 20 broth samples, and in 12 of these, one of the PCR fragments was present in a concentration at least twice as high as that of the other fragments (Table 3). This may indicate that this was the prevailing strain in that particular sample, but no isolates could be recovered to confirm this.

From two of the participating approved wild-game-handling establishments, more than one of the obtained samples were PCR-positive. When comparing the obtained MLVA profiles, the differences indicated that the contamination may have originated from different sources, possibly outside the establishment. Alternatively, the pathogens persisted in the environment or on the equipment in the facilities, potentially as biofilms [28]. MLVA may therefore be useful in the self monitoring performed by the abattoirs to identify possible sources of contamination during the slaughter process and poor hygiene routines, resulting in biofilm formation. Furthermore, in two out of the three PCR-positive samples obtained from establishment no. 6, a PCR fragment for locus V6 was present that corresponded to 31 repeats. Fragments of this size have previously not been described for this locus, and thus further studies are warranted.

The information associated with the isolates of *Y. enterocolitica* included in the present study, as well as the MLVA profiles obtained, may be used to compile a common, available database as a future reference in outbreak investigations and source attribution studies. To achieve this, the systematic implementation of MLVA in several laboratories and the sharing of data between laboratories and government agencies are needed [29]. In doing so, sporadic cases in different parts of the country could be linked and through the inclusion of data from possible sources or reservoirs, common sources of infection could be identified.

## 5. Conclusions

In the present study, similarities between isolates from humans and domestic pigs were demonstrated, as well as a perfect match in the profiles obtained for samples from rodents and pigs residing on the same farm. The results of the present study indicate that MLVA of isolates and PCR-positive enrichment broths could be a useful tool for epidemiological investigations and outbreak investigations of enteropathogenic *Yersinia* spp. However, further evaluation of the analysis of PCR-positive enrichment broths is needed. To fully utilize the potential of this method, its systematic implementation in several laboratories and common, shared databases are needed.

Further, *Y. enterocolitica* was demonstrated to be present in one third of the wild boar minced-meat samples. This is of concern for public health, and targeted information is needed both for the wild-game-handling establishments and the private hunters to reduce the risk of contamination of food.

## Figures and Tables

**Table 1:** The results from analysis of wild boar minced-meat samples obtained from approved game-handling establishments (AGHEs) and private hunters positive for the presence of *Y. enterocolitica*.

	No. of Samples	No. of Positive Samples
AGHE 1	1	0
AGHE 2	3	2
AGHE 3	1	0
AGHE 4	1	1
AGHE 5	2	0
AGHE 6	4	3
Private hunters	20	4
Total	32	10

**Table 2:** The presumptive profiles obtained in the multiple-locus variable-number tandem repeat analysis (MLVA) of *Y. enterocolitica* performed on PCR-positive enrichment broths (BHI) obtained in the analysis of wild boar minced meat originating from approved game-handling establishments (AGHEs) and from private hunters. The loci investigated were designated V2A, V4-V7 and V9, and the number of repeats of the fragments detected in each locus is specified. DNA fragments of a high concentration (i.e., more than twice that of the concentration of other fragments) is given in bold and is considered as the most likely number of repeats in that particular locus. Upon arrival to the laboratory, all samples and establishments were numbered consecutively (sample no./AGHE no.). The locations of the sampling sites for positive samples obtained from private hunters are also mapped in Figure 1.

	Locus						
Sample No., AGHE No.	V2A	V4	V5	V6	V7	V9	Presumptive Profiles
2, AGHE. 6	3	2	15	7	3	ND	3-2-15-7-3-/
			6		6		3-2-15-7-6-/
			13				
3, AGHE. 6	0	2	3	31	7	ND	0-2-3-31-7-/
			13	7	3		0-2-13-31-7-/
4, AGHE. 6	0	2	4	7	7	ND	0-2-4-7-7-/
			3	31			0-2-4-31-7-/
			9				
6, AGHE. 2	0	2	7	7	3	ND	0-2-7-7-3-/
	3		11				3-2-7-7-3-/
7, AGHE. 2	0	2	14	7	3	ND	0-2-14-7-3-/
			4				
			6				
9, Private	0	2	4	7	7	ND	0-2-4-7-7-/
			10		3		0-2-10-7-7-/
			15				0-2-15-7-7-/
11, Private	3	2	6	7	6	6	3-2-6-7-6-6
			7				3-2-7-7-6-6
29, AGHE. 4	3	2	6	7	6	6	3-2-6-7-6-6
			7				3-2-7-7-6-6
32, Private	3	2	4	7	3	6	No likely profile obtained
	0		6				
			8				
			9				
			10				
33, Private	0	2	4	7	3	3	No likely profile obtained
	3		6	31	6		
	11		8		8		
			10				

**Table 3:** The sampling location in relation to the MLVA profiles (designated A-R) obtained from 13 pure-culture isolates of *Y. pseudotuberculosis* and 14 presumptive MLVA profiles obtained from samples PCR-positive for the presence of *Y. pseudotuberculosis* originating from Swedish wild boar. The analysis of the enrichment broths yielded 12 complete presumptive MLVA profiles and 1 presumptive profile with information missing in the locus designated YPB9. The sampling locations are also mapped in Figure 1. The animals included are numbered consecutively and the tissue of origin is given. The analysis of the enrichment broth from animals no. 17 and 18 yielded two possible MLVA profiles each.

Animal Number	Sampling Location	Tissue	Origin of MLVA Profile	Profile Designation	MLVA Profile	Ref.
1	1	Left tonsil	Isolate	A ^1^	9-6-6-3-6-4-5	1 *
2	3	Right tonsil	Isolate	A ^1^	9-6-6-3-6-4-5	1
3	2	Ileoceacal lymph node	Isolate	B	6-9-12-4-5-5-5	1
4	1	Left and right tonsil	Isolate ^2^	C	3-9-7-3-2-2-5	1
5	2	Right tonsil	Isolate	C	3-9-7-3-2-2-5	1
6	4	Left tonsil	Isolate	D	6-9-5-5-6-9-6	2 **
7	5	Left tonsil	Isolate	E	2-4-8-3-2-2-4	2
8	6	Left tonsil	Isolate	F	7-5-9-4-4-6-5	2
9	6	Left tonsil	Isolate and enrichment broth	G	4-8-10-2-2-2-4	2
10	6	Left tonsil	Isolate and enrichment broth	G	4-8-10-2-2-2-4	2
11	7	Right tonsil	Enrichment broth	H	4-8-3-3-2-8-11	2
12	8	Left tonsil	Enrichment broth	I	3-10-5-6-16-4-6	2
13	5	Right tonsil	Enrichment broth	J	4-7-3-1-23-6-9	2
14	9	Right tonsil	Enrichment broth	K	3-6-3-2-6-9-8	2
		Ileoceacal lymph node	Enrichment broth	L	3-4-3-2-6-9-8	
15	10	Right tonsil	Enrichment broth	M	6-8-5-4-5-12-5	2
16	6	Left tonsil	Enrichment broth	F	7-5-9-4-4-6-5	2
17	4	Right tonsil	Enrichment broth	N	4-4-3-1-2-7-11	2
			Enrichment broth	O	4-7-3-1-2-7-11	
18	9	Right tonsil	Enrichment broth	P	9-6-5-1-6-4-5	2
			Enrichment broth	Q ^1^	9-6-5-3-6-4-5	
19	11	Right tonsil	Enrichment broth	R ^3^	10-4-15-5-4-x-5	2

^1^ Profile “A” matches with “Q” in 6 out of 7 loci, and the difference in the locus designated “YPb5” is one repeat; ^2^ in total, 4 isolates were obtained from this sample, all yielding the same MLVA profile; ^3^ incomplete profile; * Sannö et al. 2014 [21]; ** Sannö et al. 2018 [16].

## Data Availability

Data is contained within the article or Supplementary Material. The data presented in this study are available in the result section of this article and in Table S1 or upon special request from the authors.

## Supplementary Materials

The following supporting information can be downloaded at: https://www.mdpi.com/article/10.3390/ani13193055/s1, Figure S1: Example of electropherogram showing the peaks of each locus in the MLVA-analysis; Table S1: Database of isolates with MLVA-profiles, origin of isolate, year of isolation and bioserotype.
