# Supplementary figures and images for: Comparison of Multiple-Locus Variable-Number Tandem Repeat Analysis Profiles of Enteropathogenic Yersinia spp. Obtained from Humans, Domestic Pigs, Wild Boars, Rodents, Pork and Dog Food
Source: Animals (Basel). 2023 Sep 29;13(19):3055. doi: 10.3390/ani13193055 (PMC10571951; doi:10.3390/ani13193055)

1 43-13\_B05.fsa

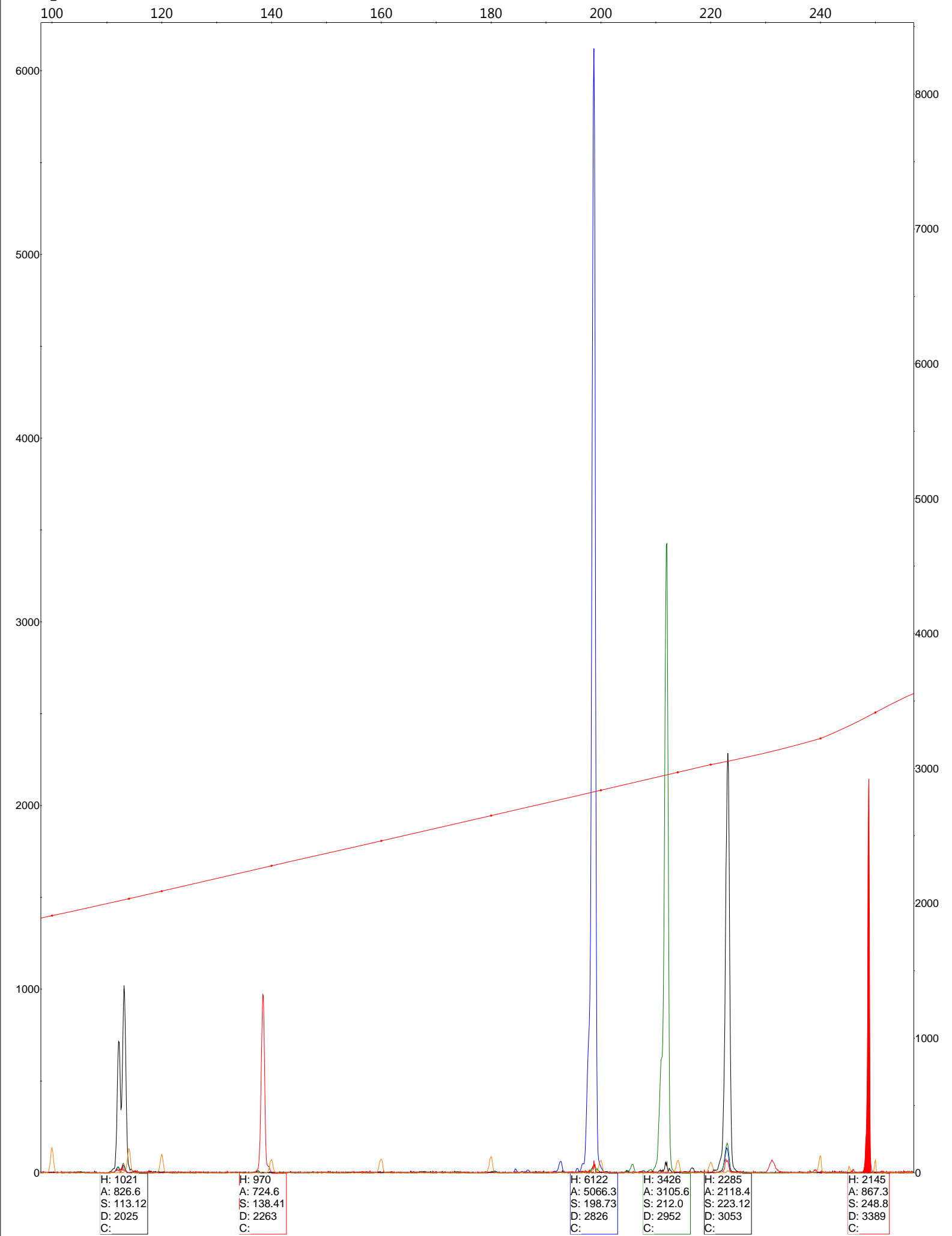

***Legend for Plot 1***

|   |                                                                                 | Legend for Plot 1                                                                 |  |     |               |
|---|---------------------------------------------------------------------------------|-----------------------------------------------------------------------------------|--|-----|---------------|
| 1 | 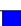   | 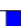   |  | 1.0 | 43-13_B05.fsa |
| 2 | 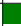  | 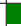  |  | 1.0 | 43-13_B05.fsa |
| 3 | 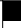 | 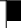 |  | 1.0 | 43-13_B05.fsa |
| 4 | 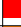 | 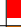 |  | 1.0 | 43-13_B05.fsa |
| 5 | 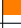 | 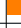 |  | 1.0 | 43-13_B05.fsa |

Supplement: Supplementary file 1 [file animals-13-03055-s001.zip › Figure S1. Example of electropherogram.pdf]
